# Supplementary material for: Preventing cation intermixing enables 50% quantum yield in sub-15 nm short-wave infrared-emitting rare-earth based core-shell nanocrystals
Source: Nat Commun. 2023 Jul 25;14:4462. doi: 10.1038/s41467-023-40031-4 (PMC10368714; doi:10.1038/s41467-023-40031-4)
Supplement: Supplementary file 1 — Supplementary Information [file 41467_2023_40031_MOESM1_ESM.pdf]

Supplementary Information

Preventing cation intermixing enables 50% quantum yield in sub-15 nm short-wave infrared-emitting rare-earth based core-shell nanocrystals

Fernando Arteaga Cardona,[1] Noopur Jain,[2, 3] Radian Popescu,[4] Dmitry Busko,[1] Eduard Madirov,[1] Bernardo A. Arús,[5, 6] Dagmar Gerthsen,[4] Annick De Backer,[2, 3] Sara Bals,[2, 3] Oliver T. Bruns,[5, 6, 7] Andriy Chmyrov,[5, 6*] Sandra Van Aert,[2, 3*] Bryce S. Richards,[1, 8*] and Damien Hudry.[1*]

[1] Institute of Microstructure Technology, Karlsruhe Institute of Technology, Karlsruhe – Germany

[2] EMAT, University of Antwerp, Antwerp – Belgium

[3] NANOlab Center of Excellence, University of Antwerp, Antwerp – Belgium

[4] Laboratory for Electron Microscopy, Karlsruhe Institute of Technology, Karlsruhe – Germany

[5] Helmholtz Pioneer Campus, Helmholtz Center Munich, Munich – Germany

[6] Functional Imaging in Surgical Oncology, NCT/UCC Dresden, Dresden – Germany

[7] Technical University Dresden, Dresden – Germany

[8] Light Technology Institute, Karlsruhe Institute of Technology, Karlsruhe – Germany

Supplementary Figures

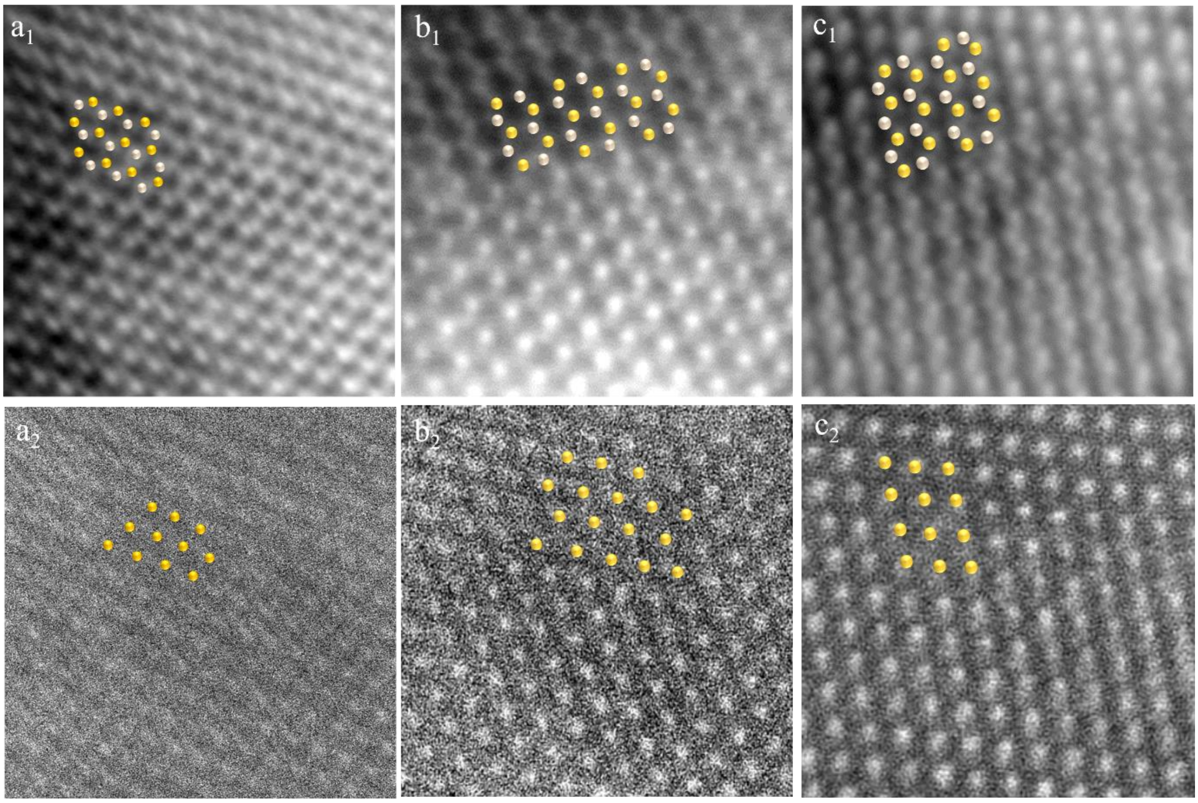

Supplementary Figure 1│ Comparison of integrated differential phase contrast (iDPC) scanning transmission electron microscopy (STEM) (top row) and high-resolution high-angle annular dark-field (HAADF)-STEM (bottom row) images. Visualization of light (fluorine atoms) and heavy (rare-earth atoms) atomic columns observed on iDPC-STEM images (top row) in pure core (a_1) nanocrystals (NCs) as well as homogeneous (b_1) and heterogeneous (c_1) core-shell NCs. The corresponding high-resolution HAADF-STEM images are shown in the panel below (a_2-c_2). The F-sites are hardly detected in the HAADF-STEM images, showing only the heavy rare-earth atoms. The heavy and light atomic columns are shown in yellow and beige, respectively.

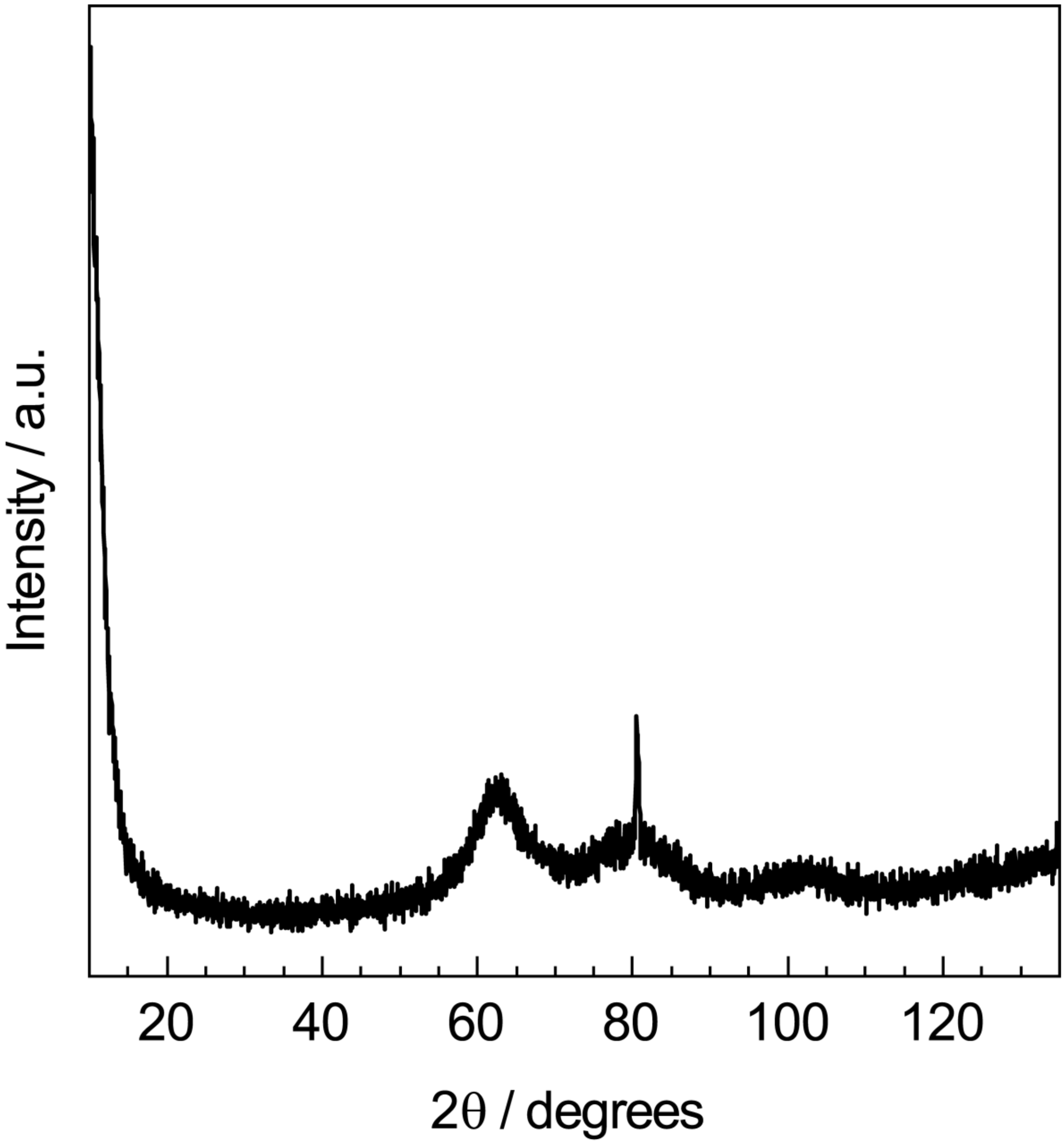

Supplementary Figure 2│ X-ray diffraction pattern of the low background (911)-oriented silicon holder. The clean (911)-oriented silicon holder exhibits the reflection at $2\theta \approx 80°$. The latter was observed on all experimental X-ray diffraction patterns acquired for core, homogeneous- and heterogeneous core-shell nanocrystals.

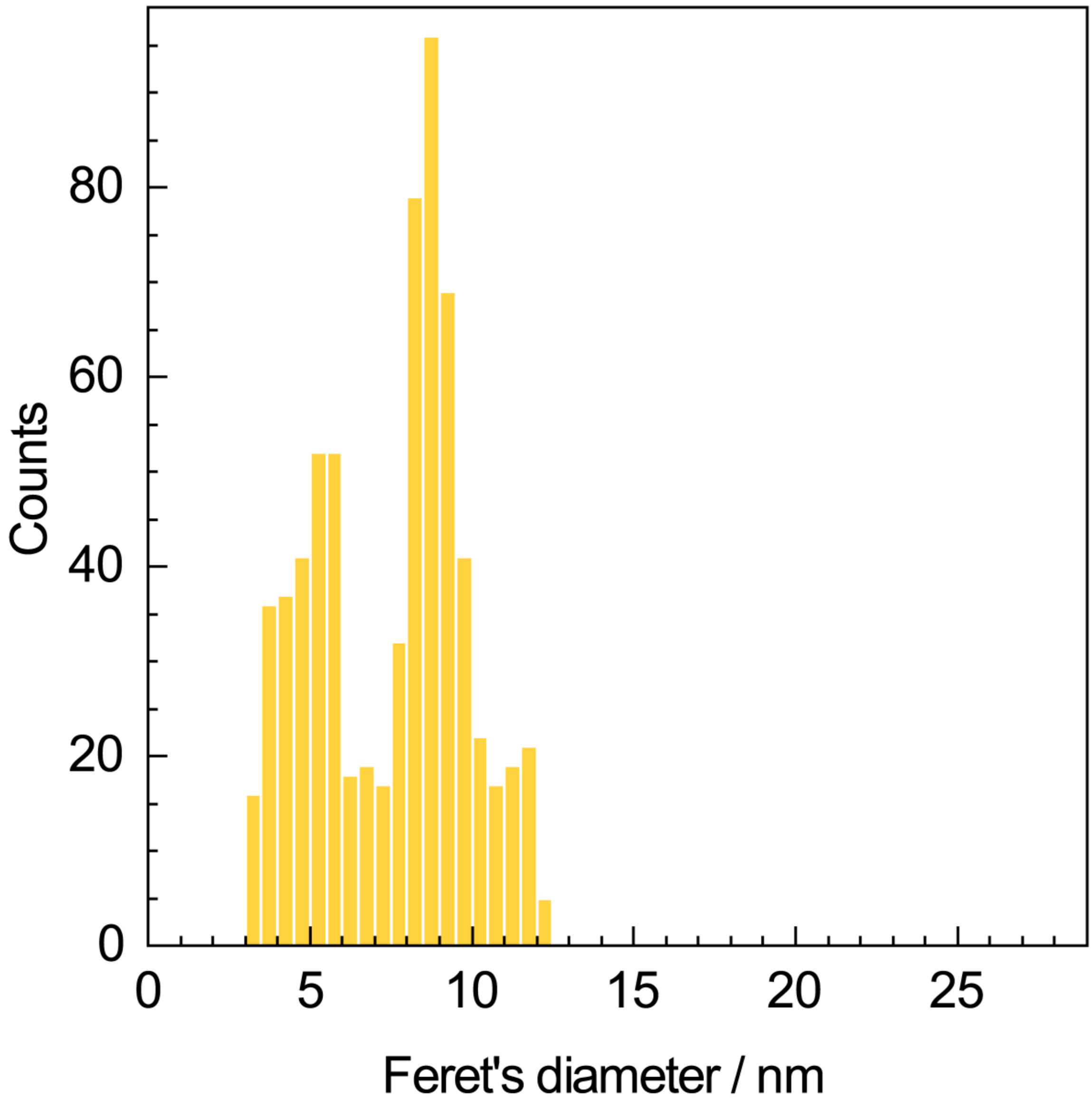

Supplementary Figure 3 | Size distribution histogram of the core region only of optically active α-$NaYF_4$:Yb:Er core nanocrystals (NCs) after growing heterogeneous CaF_2. This size distribution histogram was obtained by manually adjusting the threshold only to the bright regions observed on high-angle annular dark-field scanning transmission electron microscopy (HAADF-STEM) images of heterogeneous core-shell NCs.

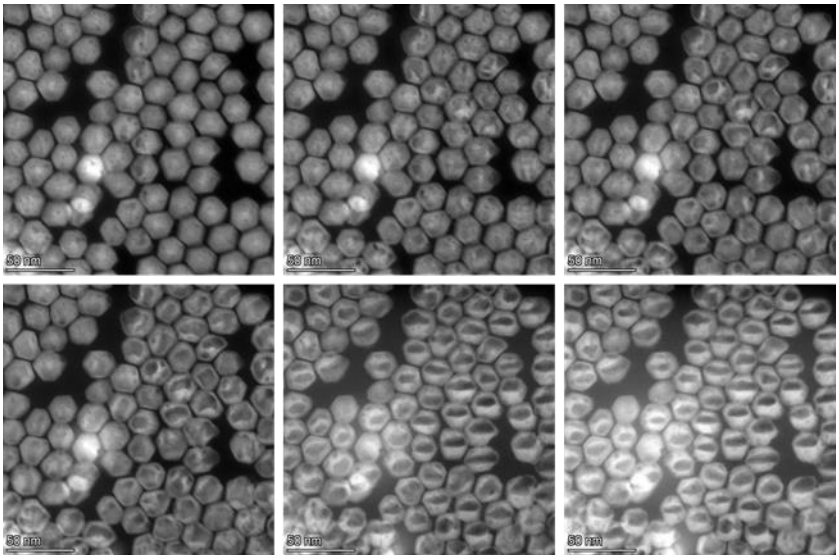

Supplementary Figure 4│ Electron beam damage during EDS acquisition. Series of high-angle annular dark-field scanning transmission electron microscopy (HAADF-STEM) images showing void formation due to electron beam exposure in homogeneous core-shell nanocrystals for a total duration of 20 min at 200 kV with a current of 1 nA. This experiment was not repeated.

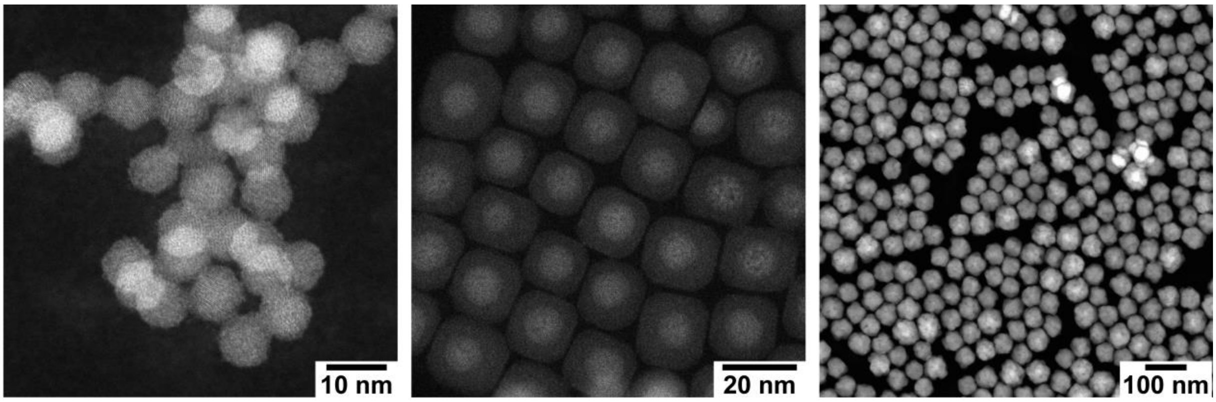

Supplementary Figure 5│ Characterization of optically active α-$NaYF_4$:Yb:Er:Ce (left) core nanocrystals (NCs) after growing heterogeneous CaF_2 (middle) and homogeneous (right) α-$NaYF_4$ shell domains. Representative high-angle annular dark-field scanning transmission electron microscopy (HAADF-STEM) images. The synthesis of core particles with Ce^{3+} doping (left) was performed three times with similar results. The growth of a heterogeneous shell domain (middle) on core particles (with Ce^{3+} doping) was performed five times with similar results. The growth of a homogeneous shell domain (right) on core particles (with Ce^{3+} doping) was performed once.

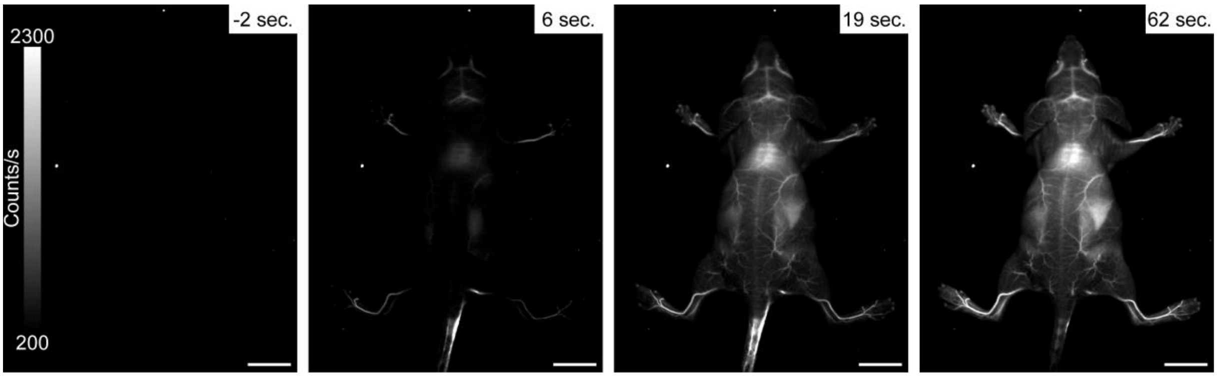

Supplementary Figure 6│ Visualization of injection dynamics for a micelle solution of heterogeneous α-NaYF4:Yb:Er:Ce@CaF_2 core-shell nanocrystals (NCs) in a mouse. Single camera frames recorded during the injection of α-$NaYF_4$:Yb:Er:Ce-CaF_2 micelles in a tail vein of a mouse. The first frame at -2 sec. time point highlights extremely low autofluorescence signal in the spectral detection window above 1100 nm upon 968 nm excitation. The bright spot located on the right-hand side of the intensity bar is outside of the mouse. No apparent accumulation of α-$NaYF_4$:Yb:Er:Ce-CaF_2 micelles in any particular organ was observed for the first 20 minutes after injection. This indicates the lower limit for the blood circulation lifetime of α-$NaYF_4$:Yb:Er:Ce-CaF_2 micelles. The determination of the exact circulation lifetime value was outside of the scope of this work. This will be investigated separately.

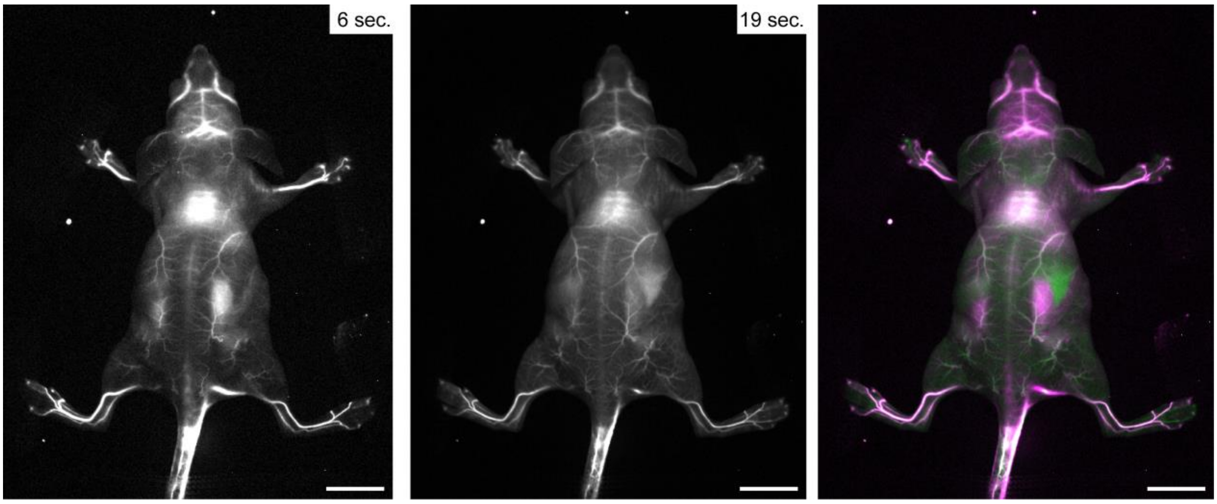

Supplementary Figure 7│ Multicolor overlap of two injection time points from Supplementary Figure E6. Two individual frames from Supplementary Figure 6 (left-hand side and middle), corrected by camera dark counts. Composite multicolour image (right-hand side) of the early injection time point (6 sec) in magenta channel and later injection time point (19 sec) in green channel.

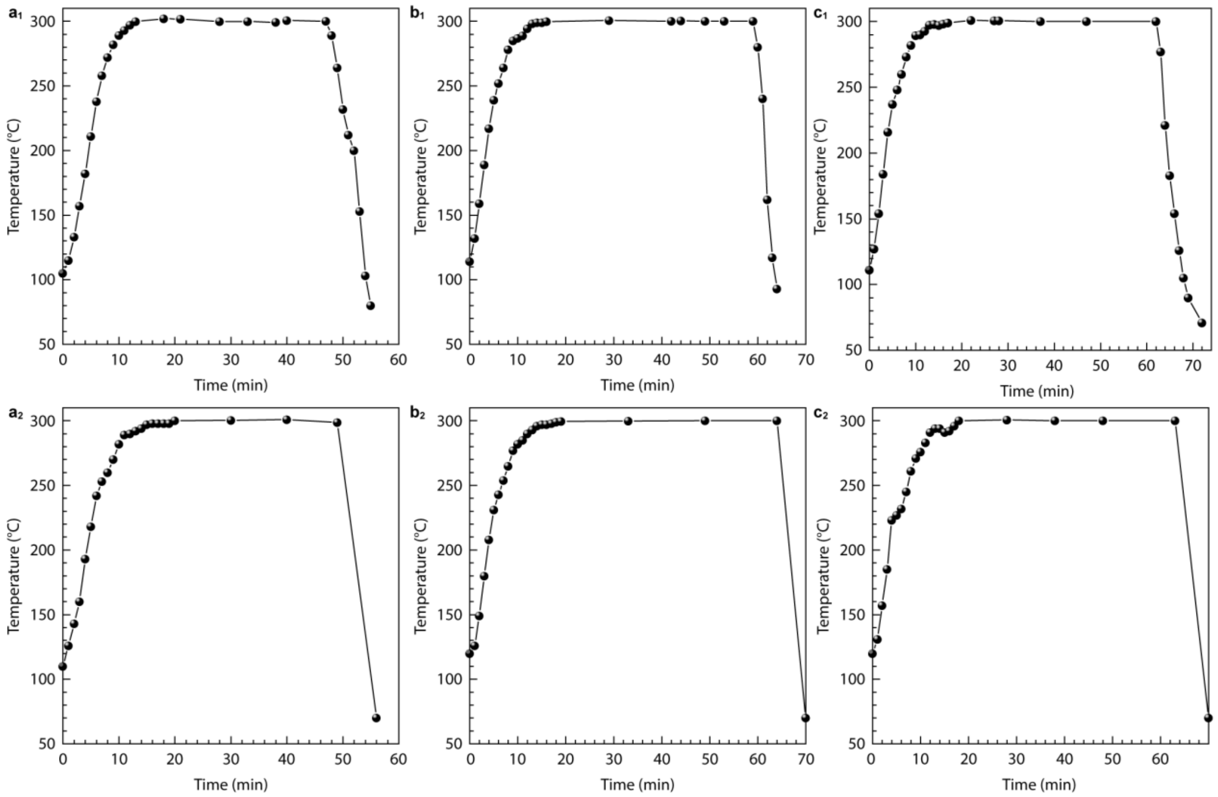

Supplementary Figure 8│ Thermal profiles recorded during the synthesis of core and core-shell nanocrystals (NCs). Experimental thermal profiles for the synthesis of core NCs (**a**) as well as homogeneous (**b**) and heterogeneous (**c**) core-shell NCs. Thermal profiles recorded for syntheses performed with Ce-undoped and Ce-doped core NCs are shown on the top and bottom rows, respectively.

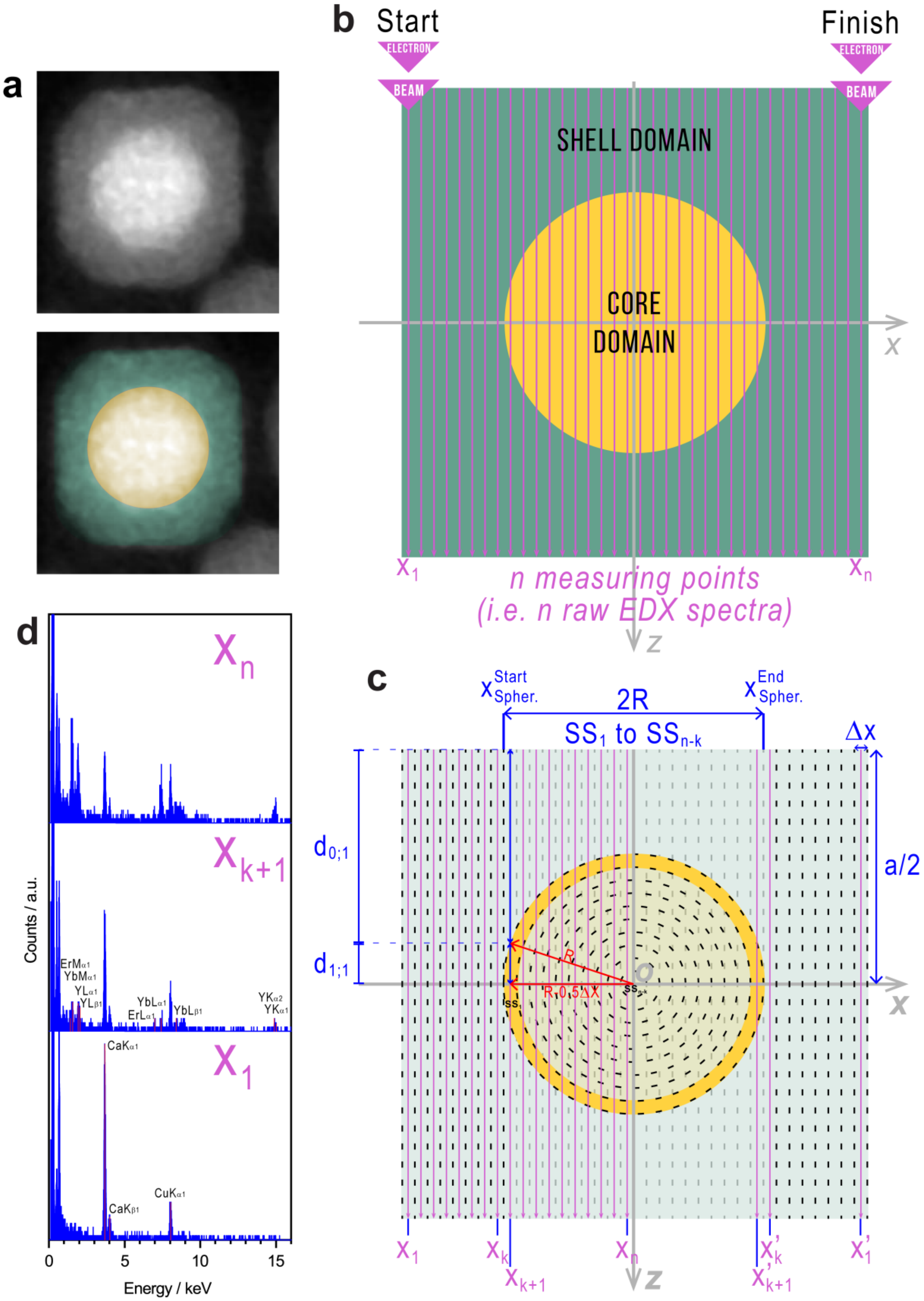

Supplementary Figure 9│ Schematic description of EDX analysis by the subshell approach. a, Representative low magnification high-angle annular dark-field scanning transmission electron microscopy (HAADF-STEM) image without (top) and with (bottom) overlaid core (orange) and shell (green) regions. **b**, Schematic description of the EDX line scan geometrical configuration showing that the raw (*i.e.* unprocessed) EDX signal is a convolution (z-direction) of various contributions depending on the spatial position along the x-direction. **c**, Mathematical subshell approach utilized to describe the individual particle described in **b**. The example shows the determination of the chemical composition of the first subshell SS_1. **d**, Example of typical raw (*i.e.* unprocessed) EDX spectra acquired for various representative spatial positions (X_1, X_{k+1}, X_n) along the x-direction. The mathematical processing of the raw EDX spectra as described in **c** enables to extract the local chemical composition for every points.

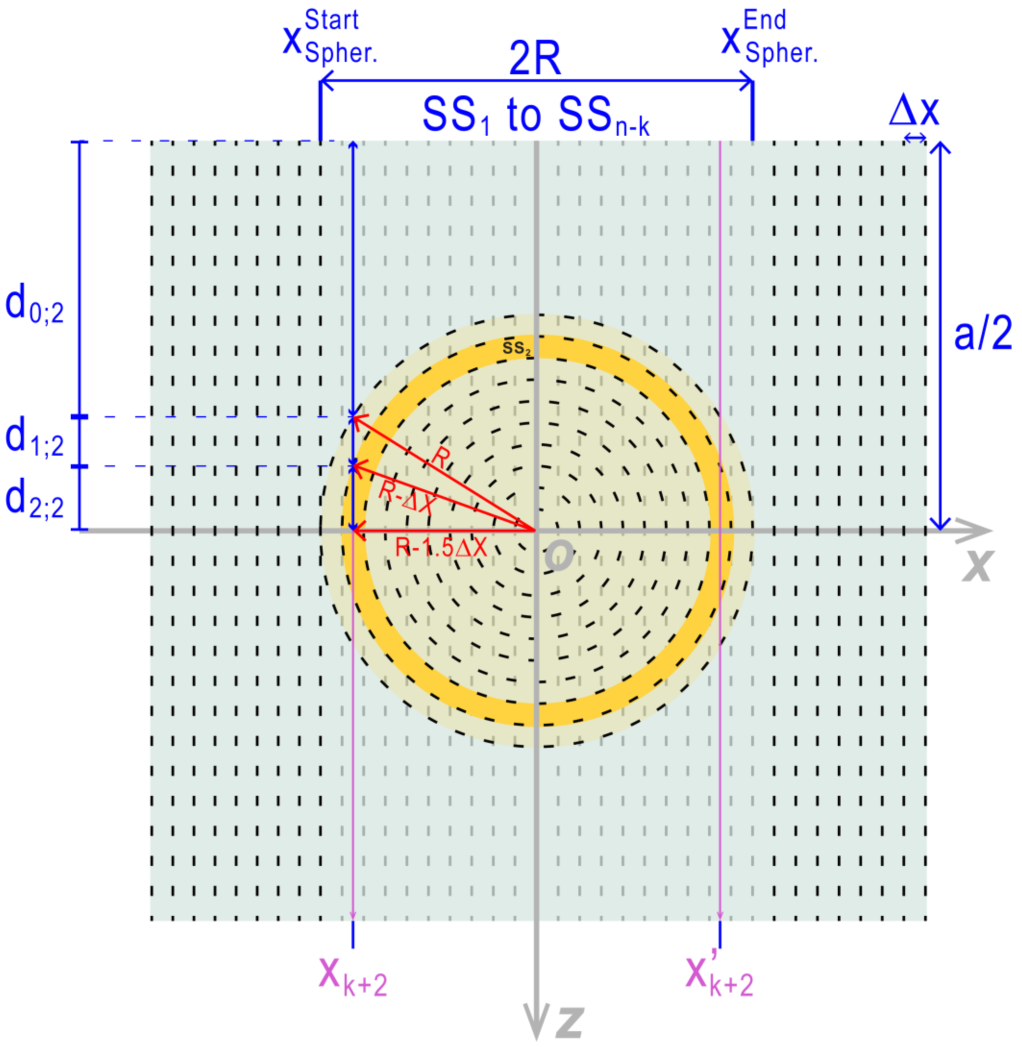

Supplementary Figure 10│ Schematic description of EDX analysis by the subshell approach. Mathematical subshell approach utilized to determine the chemical composition of the second subshell SS_2.
