# Supplementary material for: Preventing cation intermixing enables 50% quantum yield in sub-15 nm short-wave infrared-emitting rare-earth based core-shell nanocrystals
Source: Nat Commun. 2023 Jul 25;14:4462. doi: 10.1038/s41467-023-40031-4 (PMC10368714; doi:10.1038/s41467-023-40031-4)
Supplement: Supplementary file 2 — Reporting Summary [file 41467_2023_40031_MOESM2_ESM.pdf]

nature portfolio

Corresponding author(s): A. Chmyrov, S. van Aert, B.S. Richards and D. Hudry

Last updated by author(s): Jun 1, 2023

Reporting Summary

Nature Portfolio wishes to improve the reproducibility of the work that we publish. This form provides structure for consistency and transparency in reporting. For further information on Nature Portfolio policies, see our Editorial Policies and the Editorial Policy Checklist.

Statistics

For all statistical analyses, confirm that the following items are present in the figure legend, table legend, main text, or Methods section.

n/a	Confirmed	
x		The exact sample size (*n*) for each experimental group/condition, given as a discrete number and unit of measurement
x		A statement on whether measurements were taken from distinct samples or whether the same sample was measured repeatedly
x		The statistical test(s) used AND whether they are one- or two-sided<br>*Only common tests should be described solely by name; describe more complex techniques in the Methods section.*
x		A description of all covariates tested
x		A description of any assumptions or corrections, such as tests of normality and adjustment for multiple comparisons
	x	A full description of the statistical parameters including central tendency (e.g. means) or other basic estimates (e.g. regression coefficient) AND variation (e.g. standard deviation) or associated estimates of uncertainty (e.g. confidence intervals)
x		For null hypothesis testing, the test statistic (e.g. F, t, r) with confidence intervals, effect sizes, degrees of freedom and P value noted<br>*Give P values as exact values whenever suitable.*
x		For Bayesian analysis, information on the choice of priors and Markov chain Monte Carlo settings
x		For hierarchical and complex designs, identification of the appropriate level for tests and full reporting of outcomes
x		Estimates of effect sizes (e.g. Cohen's d, Pearson's r), indicating how they were calculated

Our web collection on statistics for biologists contains articles on many of the points above.

Software and code

Policy information about availability of computer code

Data collection

All x-ray diffraction pattern were acquired with a Bruker D8 Discover powder diffractometer (Bragg-Brentano geometry).
All low magnification STEM images and elemental line scans were acquired with a FEI (now Thermo Fischer Scientific) ChemiSTEM microscope.
All EDX elemental maps were acquired with a Thermo Fischer Scientific microscope equipped with a Super-X EDX system.
All atomic resolution HAADF-STEM images were acquired with an aberration corrected Thermo Fischer Scientific Titan electron microscope.
SWIR images (Figure 5) were acquired using Lightfield (Princeton Instruments, v. 6.15.1.2112).
Photoluminescence spectra and absolute quantum yields were acquired with a home made setup.
A complete description for data acquisition is given in the manuscript ("Methods" section).

Data analysis

All diffraction patterns were analyzed with TOPAS software (version 7).
All size distribution histograms were obtained with ImageJ (version 1.52a).
All line scans were quantified with the software package "TEM Imaging and Analysis" (TIA, version 4.7 SP3) combined to a home made code (Mathcad, version 14.0) to implement the subshell approach. The home made code is part of the Source Data.
All EDX chemical maps were quantified with the Thermo Fischer Scientific Velox software package (version 3.0).
The home made StatSTEM software was used to extract chemical information of individual atomic columns (atomic resolution STEM images).
The final analysis and combination of all measurements into a single PLQY intensity dependence was done in the OriginPRO data analysis software (version 2018b).
Principal component analysis for color coding of the vascular structures based on the label arrival times was done using the PoissonNMF plugin in ImageJ (version 1.53c).

For manuscripts utilizing custom algorithms or software that are central to the research but not yet described in published literature, software must be made available to editors and reviewers. We strongly encourage code deposition in a community repository (e.g. GitHub). See the Nature Portfolio guidelines for submitting code & software for further information.

Data

Policy information about availability of data

All manuscripts must include a data availability statement. This statement should provide the following information, where applicable:

- Accession codes, unique identifiers, or web links for publicly available datasets
- A description of any restrictions on data availability
- For clinical datasets or third party data, please ensure that the statement adheres to our policy

Source data are provided with this paper.

Human research participants

Policy information about studies involving human research participants and Sex and Gender in Research.

Reporting on sex and gender	Not applicable
Population characteristics	Not applicable
Recruitment	Not applicable
Ethics oversight	Not applicable

Note that full information on the approval of the study protocol must also be provided in the manuscript.

Field-specific reporting

Please select the one below that is the best fit for your research. If you are not sure, read the appropriate sections before making your selection.

[x] Life sciences   [ ] Behavioural & social sciences   [ ] Ecological, evolutionary & environmental sciences

For a reference copy of the document with all sections, see nature.com/documents/nr-reporting-summary-flat.pdf

Life sciences study design

All studies must disclose on these points even when the disclosure is negative.

Sample size	Given the exploratory nature of the experiments, no sample-size calculation was performed. The number of individuals in the animal experiment was not used to validate any biological hypothesis, but to demonstrate technological advance.
Data exclusions	None
Replication	2 mice were imaged to highlight a biological application (angiography). All attempts at replication were successful.
Randomization	No distinct groups were allocated.
Blinding	Investigators were not blinded, since no biological hypothesis was tested.

Reporting for specific materials, systems and methods

We require information from authors about some types of materials, experimental systems and methods used in many studies. Here, indicate whether each material, system or method listed is relevant to your study. If you are not sure if a list item applies to your research, read the appropriate section before selecting a response.

Materials & experimental systems

n/a	Involved in the study
☒	☐ Antibodies
☒	☐ Eukaryotic cell lines
☒	☐ Palaeontology and archaeology
☐	☒ Animals and other organisms
☒	☐ Clinical data
☒	☐ Dual use research of concern

Methods

n/a	Involved in the study
☒	☐ ChIP-seq
☒	☐ Flow cytometry
☒	☐ MRI-based neuroimaging

Animals and other research organisms

Policy information about studies involving animals; ARRIVE guidelines recommended for reporting animal research, and Sex and Gender in Research

Laboratory animals	Experiments were performed on athymic nude mice, female, 5-6 weeks old. Mice were housed at 23 °C , 45–65% humidity, with a 12/12 hour light/dark cycle.
Wild animals	The study did not involve wild animals.
Reporting on sex	The sex of the mice were not considered relevant for technology demonstration. Only female mice were used. There was no biological hypothesis test performed which required grouping by sex.
Field-collected samples	The study did not include samples collected from the field.
Ethics oversight	All ethics and protocols are in accordance with regulations of the government of Upper Bavaria.

Note that full information on the approval of the study protocol must also be provided in the manuscript.
